# Microsurgical arterial anastomosis in young and adult rats: an evolutive and comparative study

**DOI:** 10.1590/acb370604

**Published:** 2022-09-05

**Authors:** Maria Mercês Santos, Ana Cristina Aoun Tannuri, Adriana Vasconcelos Lacerda, Josiane de Oliveira Gonçalves, Luiz Roberto Schlaich Ricardi, Uenis Tannuri

**Affiliations:** 1 PhD. Universidade de São Paulo – Medical School – Pediatric Liver Transplantation Unit and Laboratory of Research in Pediatric Surgery – Pediatric Surgery Division – São Paulo (SP), Brazil.; 2 Associate professor. Universidade de São Paulo – Medical School – Pediatric Liver Transplantation Unit and Laboratory of Research in Pediatric Surgery – Pediatric Surgery Division – São Paulo (SP), Brazil.; 3 Laboratory tecnician. Universidade de São Paulo – Hospital das Clínicas – Instituto da Criança – Pediatric Liver Transplantation Unit – Pediatric Surgery Division – São Paulo (SP), Brazil.; 4 Biologist. Universidade de São Paulo – Laboratory of Research in Pediatric Surgery – São Paulo (SP), Brazil.; 5 Head professor. Universidade de São Paulo – Medical School – Pediatric Liver Transplantation Unit and Laboratory of Research in Pediatric Surgery – Pediatric Surgery Division – São Paulo (SP), Brazil.

**Keywords:** Microcirculation, Anastomosis, Surgical, Microsurgery, Blood Flow Velocity, Models, Animal

## Abstract

**Purpose:**

To evaluate the caliber of an arterial micro-anastomosis in the young growing animal using a continuous suture technique. Additionally, late morphological changes and blood flows distal to the anastomosis were evaluated.

**Methods:**

Seventy-four Wistar rats were submitted to laparotomy to access the aorta for blood flow measurement. The aorta was sectioned using microsurgery technique and an end-to-end anastomosis with continuous suture. After a period of six months to one year, the anastomosis was checked.

**Results:**

Regarding the size of the aortas, comparing the pre- and postoperative values, there was an increase of 13.33% in adult animals and 25% in young animals, without any difference in the blood flows.

**Conclusions:**

The arteries of young rats show signs of growth at the site of the anastomosis performed with continuous suture.

## Introduction

Microsurgical arterial anastomosis is a mandatory step procedure in different surgeries, including living-donor liver transplantation, a procedure indicated for the treatment of irreversible end-stage liver disease in children. The patency of this anastomosis is essential for the recipient’s survival and transplant success. In recent years, thanks to improvements in surgical techniques and pediatric intensive care, children with less than 5 kg have been increasingly submitted to surgical interventions with excellent survival outcomes, which has required a more frequent performance of microsurgical arterial anastomoses.

In general, in transplants from living donors, segments 2 and 3 of the left lobe of the liver of an adult are harvested and implanted in the recipient child after total removal of the sick liver. The graft is implanted with two venous anastomoses – hepatic vein and portal vein – and the hepatic artery anastomosis. The latter is the most critical procedure due to the reduced caliber of the artery, usually between 1 and 2 mm. Thus, the most feared and devastating complication is the postoperative occlusion of this anastomosis; its incidence rate ranges from 1 to 26%^1^-^3^, and it may have serious consequences, such as graft loss or death. Based on our cumulative experience based on 950 liver transplants in children, of which 450 were from living donors, we have standardized a simpler and more practical technique of arterial micro-anastomosis with continuous sutures after amplification of the anastomosis ends. Our results are comparable to those in the literature, with occlusion rates near 7% of the patients.

In addition, there are other questions regarding the long-term evolution of these arterial micro-anastomoses. While most patients experience long-term survival and normal growth, no experimental studies have clarified if the growth in the caliber of the arterial anastomosis is proportional to the patient’s body growth. Moreover, the potential for cicatricial stenosis, aneurysms, or late obstruction of the vessel lumen is unknown.

The objectives of the present study were to find out if the caliber of an arterial micro-anastomosis with continuous suture in a young growing animal will increase proportionately over time and to compare the changes with those observed in adult animals. We also tried to find out if, in addition to late morphological alterations, there are potential changes in the blood flow distal to the anastomosis.

## Methods

Seventy-four Wistar rats (*Rattus norvegicus albinus*, Rodentia, Mammalia) were utilized. All animals were housed under specific pathogen-free conditions, maintained in plastic cages (dimensions: 49 × 34 × 16 cm) with two littermates, on saw dust bedding, and subjected to a 12-hour light-dark cycle, in temperature 22 ± 2°C, humidity-controlled environment (55%) and free access to purified water and food (Nuvilab CR-1 commercial food, Quimtia, Colombo, PR, Brazil). Cages were changed twice a week. The animals were cared for according to the criteria outlined in the Guide for Care and Use of Laboratory Animals prepared by the National Academy of Sciences. The rats that presented clinical signs of severe pain before the end of the experimental protocol were immediately euthanized by isoflurane overdose (Isoforine^®^, Cristália, Itapira, SP, Brazil). The study protocol was reviewed and approved by the Animal Ethics Committee at our institution (Universidade de São Paulo, Medical School, São Paulo, SP, Brazil).

### Pilot experiment

To standardize the method and train the surgical team, we have operated on 20 adult rats and then 14 young rats, according to the technique described in the experimental plan. The rats went through a 24-hour acclimation period in appropriate cages with sawdust, feed, and water *ad libitum*.

### Exclusion criteria

In young animals weighing less than 90 g, the procedure was not feasible because the aortas were gelatinous and friable; therefore, we chose to only use rats weighing more than 90 g.

### Experimental procedures

In this phase, we operated on 44 adult Wistar rats aged 10 weeks and weighing between 230-260 g (females) and 360-390 g (males).

All animals were operated on by the same surgeon (MMS) and three assistants (ACT, AL, LRR). Anesthesia was induced with inhaled isoflurane followed by intramuscular ketamine hydrochloride (Ketalar) and dexmedetomidine (Precedex) at doses of 100 and 10 mg/kg, respectively. Once anesthetized, the animal was weighed and placed supine on a board lined with a heated thermal mattress, with supplemental oxygen delivered by facemask. We used microsurgery instruments and a surgical table-microscope (D F Vasconcellos S.A., Valença, RJ, Brazil) with 6-10x magnification.

The access to the aorta was done with a non-aseptic technique through a midline xiphoid-pubic laparotomy, followed by the evisceration of the intestinal loops and protection of the intestinal loops with tepid saline solution to avoid hypothermia. Blunt dissection of the abdominal aorta was performed from below the emergence of the renal arteries to the iliac bifurcation, taking care not to damage the contiguous inferior vena cava, which could jeopardize the whole procedure. The aorta was measured with a millimeter ruler; blood flow was obtained and recorded using a flowmeter. Microsurgical clamps were then placed on the aorta, and a complete linear section was performed between the proximal and distal clamps. Blood was drawn from the lumen of the aorta, and saline solution was injected, followed by end-to-end anastomosis, with continuous polypropylene 10-0 suture. After the flow was reestablished and the surgical site was checked, we could observe pulse in the aorta and the small artery immediately above the bifurcation of the aorta, i.e., above the iliac arteries (median sacral artery). The eviscerated intestinal loops were replaced into the abdominal cavity, and the abdominal wall was closed with continuous polypropylene 4-0 suture. The animals were washed in warm running water to eliminate any smell from the anesthetic or blood, and lodged in separate cages with sawdust, feed, and water *ad libitum.*

The rats were kept alive for six months to one year after surgery, and then were again anesthetized, weighed, and operated on, with a similar technique as previously described. The anastomoses were checked for macroscopic conditions and any changes such as stenosis, aneurysm, or thrombosis. The flow was again measured and recorded immediately below the anastomosis. After the data were collected, the animals were euthanized with isoflurane. Stenosis was defined as > 50% reduction in caliber compared to the vessel’s normal size. Any increase in caliber at this level was defined as an aneurysm. These criteria were utilized as an analogy to the clinical practice of pediatric liver transplantation.

After this initial phase, we started working with young Wistar rats aged 4-5 weeks and weighing between 90-120 (females) and 130-160 g (males). Thirty animals were utilized in this phase, and all the steps described for adult rats were repeated for the young rats. These animals were reoperated after six months to one year, always between 9 and 11 a.m., and the same procedures were performed as for adult animals.

To evaluate the flow in the aorta, we utilized a 1-mm ultrasound probe (Transonic Systems Inc., Ithaca, NY, United States of America), that was applied to the vessel and recorded volume flows in milliliters per minute. This probe has two transducers placed at an angle of 90° and emitting ultrasound beams, plus a reflector on the opposite side, the vessel being positioned between these two components. The objective was to obtain a direct measure of the blood flow rate^4^,^5^.

The aortic flow signals obtained from these small flowmeter transducers were transferred to an analogic-digital conversion plate via a signal conditioner, and from there to a computer. The computer records the signals using the WinDaq^®^ Lite Date Acquisition software version DI-155 and stores them using the WinDaq^®^ Recording and Playback software. All stored data were subsequently analyzed.

The blood flows were measured both in the adult and in the young animals’ groups. Pre-procedure measurements of the aortic flow were performed before the first surgery and served as a control for each animal. Approximately six months to one year after the surgical procedure, a new measurement of aortic flow was obtained distal to the anastomosis. At the end of the last experimental protocol, rats were weighed and euthanized by isoflurane overdose (Isoforine^®^, Cristália, Itapira, SP, Brazil), following the guidelines of the Ethics Committee on Animal Use of our institution. After delicate dissection, the aortas and the anastomosis sites were macroscopically examined, and the internal diameter was considered.

### Statistical analysis

All numerical values were expressed as mean and standard deviations and interquartile range. Statistical analyses were performed using the Statistical Package for the Social Sciences (SPSS) software 18.0 for Windows (SPSS, United States of America). The Shapiro-Wilk test was used to determine whether groups of data had a Gaussian distribution. The significance of between-group differences was examined using the paired t-test and Wilcoxon test. The Pearson’s χ^2^ test was used to determine if the proportion of cases with the normal flow was different among the categorical data variables. P < 0.05 (two-tailed) were considered statistically significant.

## Results

A total of 74 rats were operated on, 44 adults and 30 young animals. At the end of the experiment, it was observed that mortality rates were as follows: adults, 31.8% (n = 14); and young animals, 13.3% (n = 4).

**Figure 1:**
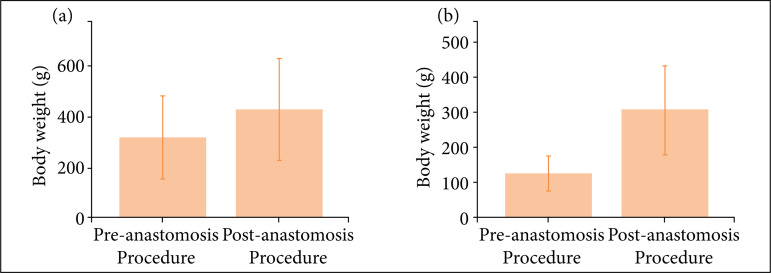
Weights of **(a)** adult and **(b)** young rats before and after the anastomosis procedure(p = 0.01 and p < 0.001, respectively).

**Figure 2:**
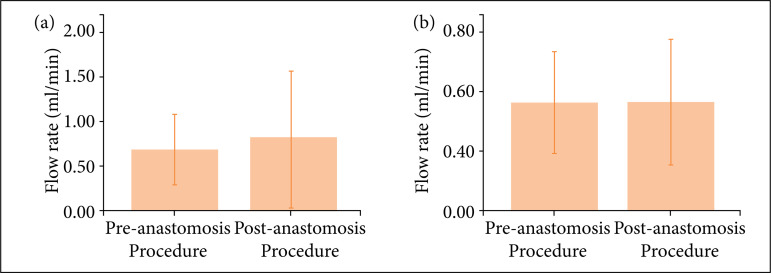
Patent and normal flows in the aorta of **(a)** adult and **(b)** young rats before andafter the anastomosis procedure (p = 0.38 and p = 0.90, respectively).

The weigh and blood flow evaluations of adult and young rats are shown in Figs. 1 and 2. It is verified that the animals presented adequate growth during the experiment.

The final results and complications are shown in Table 1. As we observe, 40% of the adult animals and 50% of the young ones presented a normal aspect of the anastomosis at the end of the experiment (Fig. 2, p = 0.45).

**Table 1:** Final results of the flows in the aorta of adult and young rats before and after the anastomosis procedure.

Age	Analysis period	Aorta Study		Flow rate (ml / min)			
			N (%)	Mean	Minimum	Maximum	SD
Adults	Pre-anastomosis procedure	Aortic flow	30 (100)	0.66	0.26	1.00	0.19
	Post-anastomosis procedure	Present and normal flow	12 (40)	0.78	0.22	2.00	0.38
		Aneurysm	6 (20)	0.80	0.80	0.80	0.00
		Stenosis	3 (10)	0.68	0.45	0.98	0.22
		Thrombosis	9 (30)	-	-	-	-
Young	Pre-anastomosis procedure	Aortic flow	26 (100)	0.47	0.30	0.73	0.11
	Post-anastomosis procedure	Present and normal flow	13 (50)	0.48	0.31	0.70	0.14
		Aneurysm	3 (11.54)	0.39	0.26	0.60	0.18
		Stenosis	3 (11.54)	0.04	0.03	0.05	0.01
		Thrombosis	7 (26.92)	-	-	-	-

SD: standard deviation.

**Figure 3:**
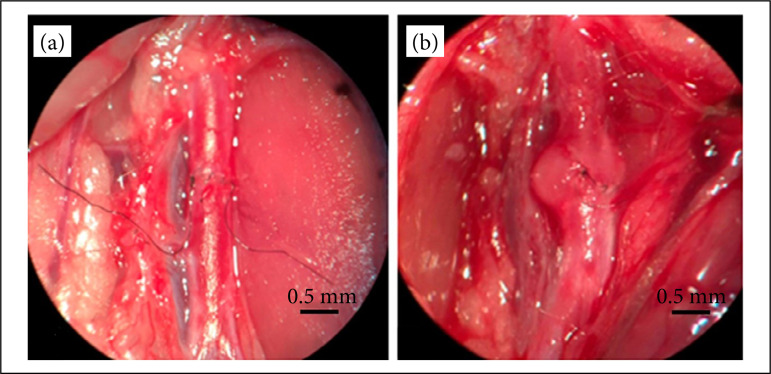
Aspects of the anastomosis. **(a)** The caliber of the aorta at the levelof the running suture. **(b)** The formation of an aneurysm at the level of theanastomosis, with a normal caliber of the distal aorta (magnification 500x).

In Fig. 3, we observed the normal anastomosis aspect and the anastomosis aspect of those animals that presented aneurysm.

Concerning the size of the aorta, the median diameter (interquartile range) in adult rats was 1.5 mm (1.2-1.8) preoperatively and 1.7 mm (1.5-2) in the late postoperative period. Therefore, in adult animals, there was a 13.33% increase in caliber compared to the pre-anastomosis measurement in adult rats (p = 0.09). In the young animals, the median diameter (interquartile range) of the aorta was 1.20 mm (1-1.50) preoperatively and 1.50 mm (1.20-1.50) in the postoperative period, corresponding to a 25% increase in the size of the aorta (p = 0.06).

As we observe, there were no statistical differences regarding thrombosis (p = 0.60), stenosis (p = 0.93), or aneurysm (p = 0.33) in young *vs*. adult rats. Finally, in the animals with arterial stenosis or thrombosis, an extensive collateral circulation around the site of occlusion was observed.

## Discussion

In growing animals, studies of arterial anastomoses have mostly been conducted in pigs and dogs. Such studies usually compare suture techniques and materials – continuous *vs*. interrupted and absorbable *vs*. non-absorbable sutures^6^.

Few studies have been conducted with growing rats due to the technical difficulties of performing micro-anastomoses in these animals. In these studies, the anastomoses were performed with interrupted sutures, and the reported results were different. Mallon *et al*.^7^ reported no significant change in the internal diameter of the arteries despite a 35% increase in the external diameter. Another study, also in growing rats, showed a 240% increase in the diameter of the anastomosis, a surprising finding, most likely due to the methodology used by the authors in the analysis. Their data were obtained after removing the arterial segment containing the anastomosis and submitting it to expansion under 150 mmHg/mm^2^-^8^, a level of pressure that is above the physiological limits.

Another unique feature of our experiment was the use of the flowmeter in the experimental phase. With this tool, it was possible to measure flow rates as low as 0.1 mL/min after performing the microvascular anastomoses. As shown in Table 1, preoperative and postoperative arterial flows in young rats remained statistically similar (0.477 ± 0.111 and 0.483 ± 0.142 mL/min,respectively). Additionally, the operated animals showed normal growth in the rear paws, without any sign of atrophy, which suggests normal blood flow to this area of the body.

Those young animals in which we found stenosis, aneurysm, or arterial thrombosis still showed normal appearance and functioning of the paws, suggesting that the complication was not acute and allowed for the establishment of collateral circulation.

Concerning flow measurement at the level of the anastomosis, a recent study has shown that blood flow values of 0.30 mL/minin the femoral artery would be indicative that the anastomosis will remain patent while values around 0.20 mL/min would indicate failure of the anastomosis^5^. The mean flow rate obtained six months after performing the anastomosis was 0.483 mL/min.The authors consider that in arterial anastomoses with a caliber between 0.6 and 1.2 mm a flow rate of 0.30 mL/min is predictive of long-term vascular patency.

Another important conclusion of the present study refers to the suture technique used for arterial micro-anastomosis. We found that a continuous suture did not prevent the growth of the vessel at the anastomosis site, due to the fact that the long continuous suture line is a spiral, not rectilinear, and it permits the arterial growth. This finding has important practical applications in microsurgery since a continuous suture is technically easier to perform than an interrupted sutures.

We also observed an 11.54% rate of stenosis of the arterial anastomosis among the young animals, similar to previous similar reports, utilizing interrupted non-absorbable sutures^8^. Other studies performed in growing pigs and dogs, using different and suture techniques reported rates of stenosis ranging from 10 to 50%^9^,^10^. In fact, based on our current results, we may conclude that the microsurgical arterial anastomosis with continuous sutures is an adequate technique.

Also worth mentioning is the formation of an aneurysm at the site of the anastomosis, which occurred in 11.54% of the animals and that resulted in reduced blood flow. Aneurysm formation is attributed to loss of continuity of the elastic lamina of the vessel wall or necrosis and fibrosis of the media after the micro-anastomosis^7^,^8^,^11^. This complication has been previously observed in other similar models, with an incidence rate between 7.5 and 41% of the anastomoses, regardless of the type of suture used^11^-^13^. The authors consider that the trauma caused by the suture needle in the middle layer of the arterial wall is an important factor in the formation of aneurysms.

## Conclusion

Our experiment suggests that, in growing rats, the arterial micro-anastomosis with the continuous suture is a feasible procedure that allows for recovery of blood flow and normal growth and had a similar incidence rate of thrombosis as found in adult animals.
